# CT Quantitative Analysis in Evaluating Type 2 Diabetes Mellitus Complicated with Interstitial Lung Abnormalities

**DOI:** 10.2174/0115734056343395250526140343

**Published:** 2025-06-03

**Authors:** Li Zhang, Qiu-ju Fan, Shan Dang, Dong Han, Min Zhang, Shu-guang Yan, Xiao-kun Xin, Nan Yu

**Affiliations:** 1 School of Medical Technology, Shaanxi University of Chinese Medicine, Xianyang, China; 2 Department of Medical Imaging, Affiliated Hospital of Shaanxi University of Chinese Medicine, Xianyang, China

**Keywords:** Type 2 diabetes mellitus, Interstitial lung abnormalities, Quantitative analysis, Lung damage, Computed tomography, Hemoglobin A1c

## Abstract

**Background:**

Type 2 diabetes mellitus (T2DM) complicated with interstitial lung abnormalities (ILAs) is often overlooked and can progress to severe diabetes-induced pulmonary fibrosis (DiPF). Therefore, early diagnosis of T2DM complicated with ILAs is crucial. Chest computed tomography (CT) is an important method for diagnosing T2DM complicated with ILAs. Quantitative computed tomography (QCT) is more objective and accurate than visual assessment on CT. However, there are currently limited studies on T2DM complicated with ILAs based on quantitative CT.

**Objective:**

This study aimed to explore the utility of quantitative computed tomography for early detection of lung injury in individuals with T2DM by examining CT-derived metrics in T2DM complicated with ILAs.

**Methods:**

We collected data from 135 T2DM complicated with ILAs on chest CT scans retrospectively, alongside 135 non-diabetic controls with normal CT findings. Employing digital lung software, chest CT images were processed to extract quantitative parameters: total lung volume (TLV), emphysema index (LAA_-950_%, the percentage of lung area with attenuation < –950 Hu to total lung volume), pulmonary fibrosis index (LAA_-700~-200_%, the percentage of lung area with attenuation from –700Hu to –200 Hu to the total lung volume), and pulmonary peripheral vascular index (ratio TAV/TNV, the number of blood vessels TNV, the cross-sectional area of blood vessels TAV). Statistical comparisons between groups utilized Mann-Whitney U or t-tests. Correlations between Hemoglobin A1c (HbA1c) levels and CT parameters were assessed via Pearson or Spearman correlations. Parameters showing statistical significance were further examined through receiver operating characteristic (ROC) analysis.

**Results:**

The T2DM-ILAs cohort displayed a significantly higher LAA_-700~-200_% compared to controls (*Z* = -7.639, *P*< 0.001), indicative of increased fibrotic changes. Conversely, TLV (*Z* =-3.120, *P*=0.002), TAV/TNV (*Z* = -9.564, P< 0.001), and LAA_-950_% (*Z* = -4.926, *P* < 0.001) were reduced in T2DM-ILAs patients. The correlation between HbA1c and various CT quantitative indicators was not significant, HbA1c and TLV (*r*=-0.043, *P*=0.618), HbA1c and TAV (*r*=0.143, *P*=0.099), HbA1c and TNV (*r*=0.064, *P*=0.461), HbA1c and LAA_-700~-200_% (*r*=0.102, *P*=0.239), HbA1c and LAA_-950_% (*r*=-0.170, *P*=0.049), HbA1c and TAV/TNV (*r*=0.175, P=0.043). The peripheral vascular marker, TAV/TNV, excelled in distinguishing T2DM-related lung changes (AUC=0.84, *P*<0.001), outperforming LAA_-700~-200_% (AUC=0.77,*P*<0.001). A composite index incorporating multiple quantitative parameters achieved the highest diagnostic accuracy (AUC = 0.91, *P*< 0.001).

**Conclusion:**

Quantitative CT parameters distinguish T2DM complicated with ILAs from non-diabetic individuals, suggesting a distinct pattern of lung injury. Our findings imply a particular susceptibility of small pulmonary blood vessels to injury in T2DM.

## 1 INTRODUCTION

Diabetes, a persistent systemic vascular disorder, Pulmonary dysfunction ranks among the frequent complications of T2DM [1]. However, lung tissue impairment, due to its abundance of capillaries and connective tissue, often manifests later than in other organs, leading to an underestimation of T2DM complicated with ILAs [2]. Consequently, research on T2DM complicated with ILAs remains sparse, with limited focus on diabetic lung pathology.

A large number of studies have shown that the main manifestations of lung damage in T2DM are pulmonary interstitial abnormalities and pulmonary microcirculation disorders [1, 3]. Chronic hyperglycemia can progress ILAs to interstitial lung disease (ILD), the most serious of which is idiopathic pulmonary fibrosis in diabetic patients [4], which can lead to a gradual decline in lung function and respiratory failure. Therefore, early diagnosis of T2DM complicated with ILAs can significantly reduce pulmonary complications caused by hyperglycemia. More and more studies have shown that some anti-diabetic drugs have anti-fibrotic effects [5, 6]. Early detection of T2DM complicated with ILAs can also help to intervene and guide treatment promptly.

The 2020 Fleischner Society consensus [7] defines ILAs as radiological observations, typically incidentally detected during CT scans in individuals without suspected interstitial lung disease. Currently, the diagnosis and evaluation of ILAs in patients with T2DM mainly rely on chest CT scans [8]. For the assessment of lesions, subjective visual evaluations are easily influenced by subjective consciousness and diagnostic experience, and there are easily differences among and within observers, lacking reliability [9]. Therefore, there is an urgent need for a more objective and accurate method to evaluate ILAs.

Quantitative CT mainly uses the density masking method, which is a method that uses computer algorithms to measure the density in Hounsfield units (Hu) of each voxel in the lung parenchyma during a CT scan [10]. Using this technique, density thresholds for normal lungs and abnormal features can be defined to determine the percentage of lungs in the region of interest [9]. In addition, it can also be combined with lung segmentation algorithms to automatically measure the diameter, area, and number of tracheal blood vessels, providing quantitative indicators that will help improve the ability to diagnose and evaluate lesions in multiple aspects [11].

## 2 MATERIALS AND METHODS

**Fig. (1):**
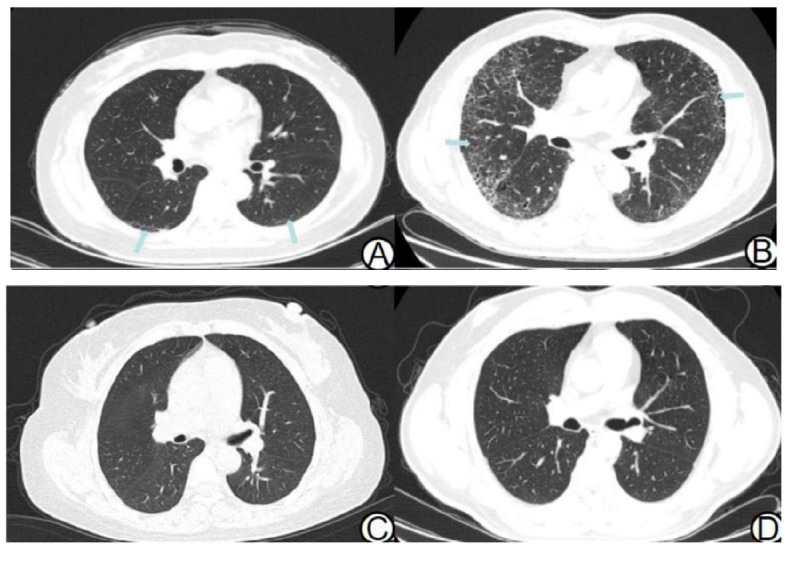
Chest CT images of two patient groups. CT scans of patients from the T2DM-ILAs group (**A** and **B**). CT scans of patients from the control group (**C** and **D**).

This retrospective investigation was ethically sanctioned by our institutional review board, waiving the requirement for individual patient consent. We compiled HbA1c and CT data from 135 patients diagnosed with T2DM complicated by ILAs at our institution from January 2022 to January 2023. Inclusion criteria comprised: (1) a confirmed diagnosis of T2DM adhering to the 2022 American Diabetes Association Diabetes Medical Diagnosis and Treatment Standards [12]; (2) the performance of a chest CT scan upon hospital admission; and (3) the identification of ILAs on chest CT scans, evaluated by two radiologists with a minimum of five years experience, consistent with the 2020 Fleischner Society Consensus [7]. ILAs were identified as gravity-independent pulmonary interstitial alterations detected incidentally by CT, covering at least 5% of the lung area. Imaging characteristics included ground-glass or reticular patterns, traction bronchiectasis, honeycombing, and non-emphysematous cysts (Fig. **1**). Patients were excluded based on the following: (1) age younger than 20 or older than 65 years; (2) incomplete medical records or CT imaging data; (3) familial history of interstitial lung diseases; (4) presence of other established interstitial lung diseases or a background of connective tissue diseases; and (5) conditions impeding image segmentation, including lung malignancies, hemangiomas, pleural effusions, or a history of thoracic surgery. In addition, 135 non-diabetic physical screening subjects with no abnormal changes in chest CT served as the control group, and their exclusion criteria were the same as those in the T2DM-ILAS group. To avoid the influence of age and gender, the age interval of the control group was selected according to the age interval of the T2DM-ILAs group, and the ratio of male to female in the control group was selected according to the ratio of male to female in T2DM-ILAs group.

### 2.1 Chest CT Acquirement

GE Discovery 750 HD (GE Discovery 750 HD, Xi'an, China) was used to obtain chest CT images. Non-contrast-enhanced CT scanning was performed. Before the scan, patients received instructions on proper breathing techniques. During the scan, patients lie supine with raised arms and an end-inspiratory breath-hold scan. The scanning spanned from the lung apex to the lung base, utilizing the following parameters: tube voltage of 120 kV, tube current of 200 mA, imaging matrix of 512×512, a scanning slice thickness of 5.0 mm, a pitch of 1.375:1, and the tube rotation time 0.5s/r. Reconstruction utilized a standard kernel, with a reconstruction slice thickness and interval both set at 1.25 mm. The lung window width was 1200 Hu, and the window level was -600 Hu.

### 2.2 Subjective Evaluation

Two experienced radiologists, each with over five years of expertise, independently reviewed the chest CT scans. They identified imaging characteristics such as ground-glass or reticular patterns, encompassing interlobular and interstitial thickening, bronchial vascular bundle thickening, subpleural lines, traction bronchiectasis, honeycombing, and non-emphysematous cysts. These abnormalities affected more than 5% of any lung zone [7]. In instances of disagreement between the two radiologists, a third senior radiologist, possessing 10 years of experience in chest CT interpretation, made the final judgment.

### 2.3 Quantitative CT Parameters Acquisition

The inspiratory CT scan data were processed using the Digital Lung platform (Dexin-FACT, Xi'an, China). The methodology followed previous literature^.^ Here's a breakdown of the specific measurements:

1. Total lung volume (TLV): The software employed a three-dimensional adaptive boundary marching algorithm to derive comprehensive lung data and automatically compute the TLV. Detailed descriptions of computerization solutions have been reported elsewhere [13].

2. Emphysema index: At present, CT mainly uses the density masking method for quantitative measurement of emphysema, that is, a CT threshold is set. When the density of the emphysema area is lower than this threshold, the percentage of emphysema area in the total lung volume can be quantitatively displayed, that is, low attenuation area percentage (LAA%) [14]. The CT threshold for inspiratory LAA% is usually set at -950 Hu [15].

3. Pulmonary fibrosis index: The LAA_-700~-200_% value represented the percentage of the lung area with attenuation from -700 to -200 Hu to the total lung volume. This measurement, obtained from the lung density histogram's automatic measurement module, serves as the pulmonary fibrosis index. The software determines the lung percentage of the region of interest by defining density thresholds for normal lung and abnormal lung features. Previous studies have reported that the area between -700 Hu and -200 Hu reflects fibrosis in the lung [10, 11, 16].

4. Pulmonary peripheral vascular index: It is divided into two steps. The first step is the segmentation of bronchi and pulmonary vessels. Previous research reports have demonstrated that differential geometry methods can be used to automatically segment bronchi and vessel trees. The segmentation algorithm includes anatomical structure modeling and principal curvature calculation. Differential geometry methods are also used to segment the airway tree to locate cross sections [16-18]. The above method was used by the software to automatically segment the bronchial and pulmonary vascular structures and generate three-dimensional images of these structures. The final segmentation results were reviewed and verified by 2 radiologists. If the computerized algorithm could not accurately identify the airway tree, then these CT datasets were excluded from further quantitative analysis of the pulmonary peripheral vascular. The second step is to obtain the pulmonary peripheral vascular index based on the segmented bronchial vascular tree [11]: Specifically, the bronchus with a diameter of 2 mm was selected from the lower lobes of both lungs to obtain the number and cross-sectional area of blood vessels in the visible area. The software's vascular automatic measurement module quantified the number and cross-sectional area of these peripheral vascular within an 80 mm2 viewing area. To avoid physiological anatomy variations, the cross-sectional area-to-number ratio of 2mm peribronchial vessels in both lower lobes was calculated.

### 2.4 Statistical Analysis

GraphPad Prism 9.0 software and SPSS 26.0 were used for graphical display and statistical analysis of data. The Kolmogorov-Smirnov test was used to test the normality of the measurement data. The measurement data that conformed to the normal distribution were expressed as (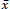
± s). Non-normally distributed measurement data are presented as median with upper and lower quartiles. The measurement data conforming to normal distribution were analyzed by independent two-sample *t t*est, and the measurement data not conforming to normal distribution were analyzed by the Mann-Whitney *U* test. For categorical data comparisons between groups, the χ^2^ test was employed. Pearson correlation (normal distribution) or Spearman rank correlation (non-normal distribution) were used to analyze the correlation between HbA1c and quantitative parameters of lung CT. The discriminative ability of the quantitative CT indices was assessed using the area under the curve (AUC) from receiver operating characteristic (ROC) analyses. A *P* value less than 0.05 denotes statistical significance.

## 3 RESULTS

### 3.1 Characteristics of the Patients

In the T2DM-ILAs group, 75 males and 60 females; median [IQR] age, 53 (49, 58) years, with disease durations spanning 0.16 to 28 years; notably, 59 cases exceeded 5 years. Their fasting blood glucose median 10.15 [IQR, 8.00, 13.05] mmol/l, and HbA1c median was 8.60% [IQR, 7.30%, 10.30%]. Similarly, the control group also had 75 males and 60 females, median [IQR] age, 53 (46, 56) years. No significant differences were observed in age or gender distribution between the groups. (*Z*=-1.600,*P*=0.110;χ^2^=0.000,*P*=1.000).

### 3.2 Subjective Evaluation Results

In both the T2DM-ILAs and control groups, a subjective evaluation was conducted. Among the 135 patients with T2DM-ILAs, CT scans revealed ground-glass shadows in 13 cases (9.63%), interlobular septal thickening in 14 cases (10.37%), subpleural lines in 49 cases (36.29%), thickening of bronchial vascular bundles in 58 cases (42.96%), and interlobular interstitial thickening in 4 cases (2.96%);4 cases (2.96%) showed thickening of interlobular septal thickening and subpleural lines simultaneously. However, there were no indications of honeycomb shadows, traction bronchiectasis, or non-emphysematous cysts in this cohort. Typically, in T2DM-ILAs patients, chest lesions predominantly appear in the lower lung regions. In contrast, the chest CT scans of all 135 patients in the control group were normal (Table **1**).

### 3.3 Quantitative CT Parameters Results

The quantitative CT parameter measurements for both the T2DM-ILAs group and the control group are depicted in Figs. (**2** and **3**). Comparative results are summarized in Table **2**. The LAA_-700~-200_% value was notably higher in the T2DM-ILAs group than in the control group (*Z*=-7.639, *P*=0.000). Conversely, the TLV, TAV/TNV, and LAA_-950_% values in the T2DM-ILAs group were notably lower than those in the control group (*Z* = -3.120, *P* = 0.002; *Z* = -9.564, *P* = 0.000; *Z* = -4.926, *P*= 0.000).

### 3.4 Correlation Analysis Results

**Fig. (4):**
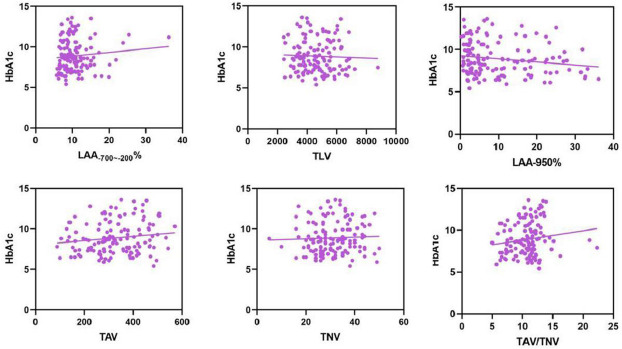
Correlation analysis results.

The correlation between HbA1c and various quantitative CT parameters was not significant. HbA1c and LAA_-950_% (*r*=-0.170, *P*=0.049), HbA1c and TAV/TNV (*r*=0.175, *P*=0.043), HbA1c and LAA_-700~-200_% (*r*=0.102, *P*=0.239), HbA1c and TLV (*r*=-0.043, *P*=0.618), HbA1c and TAV (*r*=0.143, *P*=0.099), HbA1c and TNV (*r*=0.064, *P*=0.461) (Fig.**4**).

### 3.5 Analysis of the Diagnostic Value of Quantitative CT Parameters

**Fig. (5):**
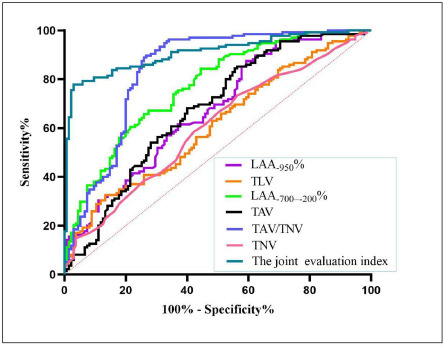
ROC curves of quantitative CT parameters for Diagnosis of T2DM-ILAs.

The ROC curve assessed the efficacy of each parameter and the joint evaluation index constructed with multiple quantitative parameters in identifying interstitial lung abnormalities in T2DM patients (Table **3**, Fig.**5**). The pulmonary peripheral vascular index TAV/TNV demonstrated an AUC of 0.84 (*P* < 0.001), while the pulmonary fibrosis evaluation index LAA_-700~-200_% had an AUC of 0.77 (*P*< 0.001). The joint evaluation index constructed with multiple quantitative parameters (LAA_-950_%, TLV, LAA_-700~-200_%, TAV/TNV, TAV and TNV) had the best identification ability (AUC=0.91, *P* < 0.001).

## 4 DISCUSSION

Given the lung's extensive alveolar capillary network, it's susceptible to microvascular damage, positioning it as a primary target organ for diabetes-related complications. This primarily manifests as interstitial lung abnormalities [3, 19, 20]. The main means of evaluating interstitial lung abnormalities is chest CT, and visual assessment of the severity of the lesions alone is not sufficient. Quantitative CT offers objectivity and precision in discerning and assessing interstitial lung abnormalities [21]. Leveraging lung density histograms to quantitatively gauge compartments like ground glass, reticular abnormalities, and fibrotic tissue relative to total lung volume ensures reproducibility and aligns with pathological observations [22-24]. Consequently, our study quantitatively analyzed CT images from T2DM patients, revealing a notably heightened level of pulmonary fibrosis compared to the control group. T2DM patients show higher levels of fibrosis, which may be related to the fact that long-term high blood sugar leads to the release of a large number of pro-inflammatory factors, stimulates the activation of inflammatory cells, abnormal repair of alveolar epithelial cells, secretes a large amount of extracellular matrix, and accelerates the formation of pulmonary fibrosis [25]. It may also be related to the imbalance of oxidation and antioxidants induced by high blood sugar, which promotes lung epithelial damage and fibrosis [4].

In this study, it was found that the TLV of T2DM patients was reduced compared with the control group, and the reason for the reduction of TLV in T2DM patients was related to the reduced lung compliance. The mechanism of reduced lung compliance caused by T2DM is that in the condition of high glucose, the non-enzymatic glycosylation of collagen weakens the degradation of collagen, and the accumulation of type 4 collagen weakens the elasticity of the lung, thus leading to a decrease of lung elasticity and compliance [26].

T2DM patients complicated with ILAs may also exhibit concurrent emphysema. Our evaluation using LAA_-950_% indicated that the emphysema index LAA_-950_% in the T2DM group was significantly lower than that in the control group. LAA_-950_% is a quantitative lung CT index obtained by scanning reconstruction in the state of full inspiration. It can be inferred that T2DM patients complicated with ILAs are more prone to poor inspirations. This may be due to oxidative stress and chronic inflammation caused by persistent hyperglycemia, which damages type II alveolar epithelial cells, thereby reducing the secretion of alveolar surface active substances, atrophy, and even collapse of alveoli, resulting in reduced lung gas retention and reduced value of LAA_-950_% [27].

Pulmonary microangiopathy stands as a principal manifestation of diabetic lung injury. The oxidative-antioxidative imbalance, driven by hyperglycemia and chronic inflammation, significantly contributes to pulmonary endothelial damage and fibrosis. This manifests chiefly as thickened basement membranes in the alveolar septum, walls, and capillaries, diminishing pulmonary microvascular volumes and exacerbating peripheral vessel alterations [3, 28]. Given that pulmonary blood vessels align with bronchi to shape the lung interstitium, targeting respiratory bronchioles aids in effectively pinpointing peripheral vessels. In our investigation, we selected respiratory bronchioles (2mm diameter) in both lower lung lobes to quantify surrounding blood vessel cross-sectional areas and counts. Corrective measures were applied using vessel counts to mitigate physiological anatomy. Results demonstrated a notable reduction in peripheral blood vessel lumen area in the T2DM group, aligning with pathological shifts [29].

Currently, diabetic lung injury imaging evaluations predominantly rely on visual assessments, susceptible to various factors, including observer variability. In contrast, quantitative CT offers a more objective and precise depiction of lung alterations, proving invaluable for early detection of T2DM complicated with ILAs. ROC curve analyses underscored the utility of the peripheral vascular index TAV/TNV (AUC = 0.84) and the pulmonary fibrosis index LAA_-700~-200%_ (AUC = 0.77) as superior markers for identifying T2DM complicated with ILAs. Moreover, the joint evaluation index constructed with multiple quantitative parameters had the best identification ability (AUC=0.91).

Persistently high blood sugar levels may alter inflammatory pathways associated with impaired lung function. Oxidative stress and non-enzymatic glycation of proteins are recognized in the etiology of diabetic lung injury [30]. Literature has shown that the increase of HbA1c is related to the decrease of lung function, such as Forced vital capacity (FVC), and the control of HbA1c level helps to delay the development of lung lesions [31]. Therefore, this study analyzed the correlation between HbA1c and various quantitative indicators, and found that there was no significant correlation between HbA1c and quantitative parameters of lung CT. The reason for this result may be due to the mild lung lesions in the T2DM patient group selected in this study, as well as the strong compensatory capacity of the lungs. Generally, abnormal lung function occurs only when the lung injury is greater than 30% [32].

## 5 LIMITATIONS

In this study, quantitative CT provided insights into lung changes in T2DM complicated with ILAs. However, several limitations warrant acknowledgment. Firstly, we only analyzed the changes in lung imaging in patients with T2DM, and did not combine clinical pulmonary function tests to study the functional changes in the lungs. The main reason is that this study is retrospective, and the data is not perfect. However, it is necessary to study the relationship between changes in lung function and blood sugar, so this is also the direction of subsequent research. Secondly, this study may be affected by some potential confounding factors, such as the lack of stratified analysis between men and women, which may have affected the ROC results. Lastly, we only analyzed the inspiratory phase CT quantitative parameters, and CT quantitative parameters in this study did not apply to the analysis of expiratory phase CT. Conventional clinical use of quantitative CT is limited by slice thickness, which may lead to the loss of some very early ILAs.

## CONCLUSION

In conclusion, quantitative CT is a key tool for diagnosing and evaluating lung lesions in patients with T2DM. ILAs can progress to DiPF, which has the worst prognosis. The identification and management of T2DM complicated with ILAs can help prevent or delay the occurrence of DiPF and improve the quality of life of patients. Some anti-diabetic drugs have anti-fibrosis effects. Quantitative CT is used to regularly monitor the degree of fibrosis and small vessel injury in T2DM patients complicated with ILAs, which is helpful to guide the drug treatment of T2DM patients, delay the progressive decline of lung function, and prevent or delay the occurrence of respiratory failure.

## Figures and Tables

**Fig. (2):**
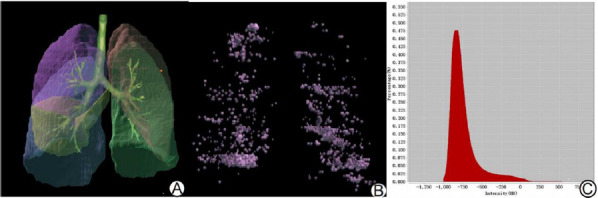
Schematic diagram of quantitative CT parameters. Adaptive boundary method for acquiring whole lung volume (TLV) (**A**). Lung density visualization for determining LAA_-950_% (**B**). Calculation of LAA_-700~-200_% using the lung density histogram (**C**).

**Fig. (3):**
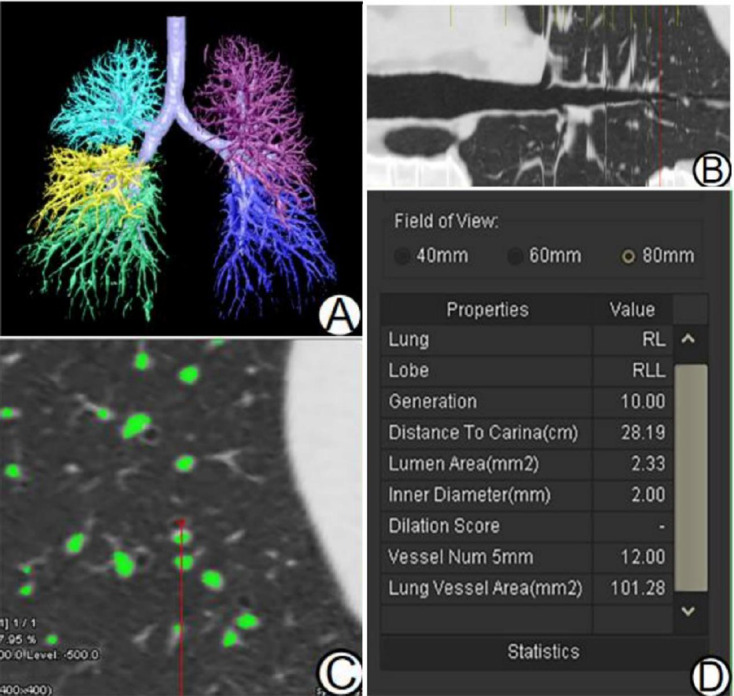
Pulmonary peripheral vascular index measurements and results. Segmentation and reconstruction of the bronchial vascular tree based on differential geometry methods (**A**). Diagram illustrating the reconstruction curve of the bronchus. The extended red line indicates the location of a bronchus with a 2 mm inner diameter (**B**). An 80 mm2 visual area shows peripheral blood vessels, with the green section representing pulmonary blood vessels (**C**). Results from the measurement of peripheral vascular (**D**).

**Table 1:** Subjective evaluation results.

Visual Assessment	T2DM-ILAs Group(n=135)	Control Group (n=135)
Ground-glass	13(9.63%)	0
Interlobular septal thickening	14(10.37%)	0
Interlobular interstitial thickening	4(2.96%)	0
Thickening of bronchial vascular bundles	58(42.96%)	0
Subpleural lines	49(36.29%)	0
Traction bronchiectasis	0	0
Honey combing	0	0
Non-emphysematous cysts	0	0

**Abbreviation:**T2DM-ILAs group: T2DM complicated with ILAs group.

**Table 2:** Quantitative CT parameters results between T2DM-ILAs and the control group.

Quantitative CT Parameters	T2DM-ILAs Group (n=135)	Control Group (n=135)	*Z/t*	*P*
LAA_-950_%	3.33(1.45,7.81)	6.29(2.65,16.92)	-4.926	0.000
TLV/mL	4061.57(3236.11,4861.53)	4503.43(3712.78,5452.10)	-3.120	0.002
LAA_-700~-200_%	12.78(10.18,17.99)	9.13 (7.37,11.31)	-7.639	0.000
TNV	34.21±8.67	31.00±8.34	3.104	0.002
TAV (mm^2^)	263.31±87.86	327.24±108.62	-5.317	0.000
TAV/TNV	7.83(6.59,8.65)	10.95(9.02,12.05)	-9.564	0.000

**Notes**: T2DM-ILAs group: T2DM complicated with ILAs group.LAA_-950_%: the percentage of lung area with attenuation < –950 Hu to total lung volume; TLV: total lung volume; LAA_-700~-200_%: the percentage of lung area with attenuation from –700 Hu to –200 Hu to the total lung volume; TAV: the cross-sectional area of blood vessels; TNV: the number of blood vessels.

**Table 3:** Analysis of the diagnostic value of quantitative CT parameters.

quantitative CT parameters	AUC	95%CI	*P*	Cutoff	Sensitivity		Specificity
LAA_-950_%	0.67	0.610~0.737	<0.001	9.90	0.87	0.41	
TLV	0.61	0.543~0.677	0.0018	3423	0.33	0.87	
LAA_-700~-200_%	0.77	0.713~0.823	<0.001	9.51	0.84	0.56	
TAV/TNV	0.84	0.787~0.887	<0.001	9.29	0.89	0.74	
TAV (mm^2^)	0.67	0.610~0.738	<0.001	345.90	0.85	0.44	
TNV	0.60	0.535~0.669	0.0038	28.50	0.73	0.44	
The joint evaluation index	0.91	0.872~0.945	<0.001	0.69	0.97	0.78	

**Notes**: T2DM-ILAs group: T2DM complicated with ILAs group.LAA_-950_%: the percentage of lung area with attenuation < –950 Hu to total lung volume; TLV: total lung volume; LAA_-700~-200_%: the percentage of lung area with attenuation from –700 Hu to –200 Hu to the total lung volume; TAV: the cross-sectional area of blood vessels; TNV: the number of blood vessels.

## Data Availability

The authors confirm that the data supporting the findings of this study are available from the corresponding author [N.Y], upon a reasonable request.

## LIST OF ABBREVIATIONS

T2DM: Type 2 Diabetes Mellitus
ILAs: Interstitial Lung Abnormalities
DiPF: Diabetes-induced Pulmonary Fibrosis
CT: Computed Tomography
QCT: Quantitative Computed Tomography
LAA_-950_%: The percentage of lung area with attenuation < –950 Hu to total lung volume
LAA%: Low Attenuation Area percentage
TLV: Total Lung Volume
LAA_-700~-200_%: The percentage of lung area with attenuation from –700 to –200 Hu to the total lung volume
TAV: The cross-sectional area of blood vessels
TNV: The number of blood vessels.
HbA1c: Hemoglobin A1c
ILD: Interstitial Lung Disease
Hu: Hounsfield units
AUC: Area Under Curve
ROC: Receiver Operating Characteristic
FVC: Forced vital capacity

## STANDARDS OF REPORTING

STROBE guidelines were followed.
